# Which Bone Marrow Sparing Strategy and Radiotherapy Technology Is Most Beneficial in Bone Marrow-Sparing Intensity Modulated Radiation Therapy for Patients With Cervical Cancer?

**DOI:** 10.3389/fonc.2020.554241

**Published:** 2020-12-17

**Authors:** De-Yang Yu, Yan-Ling Bai, Yue Feng, Le Wang, Wei-Kang Yun, Xin Li, Jia-Yu Song, Shan-Shan Yang, Yun-Yan Zhang

**Affiliations:** ^1^ Department of Radiation Physics, Harbin Medical University Cancer Hospital, Harbin, China; ^2^ Department of Gynecological Radiotherapy, Harbin Medical University Cancer Hospital, Harbin, China

**Keywords:** cervical cancer, bone marrow sparing, helical tomotherapy (HT), volume-modulated arc therapy (VMAT), intensity-modulated radiotherapy (IMRT)

## Abstract

**Background:**

To evaluate the dosimetric parameters of different bone marrow sparing strategies and radiotherapy technologies and determine the optimal strategy to reduce hematologic toxicity associated with concurrent chemoradiation (cCRT) for cervical cancer.

**Methods:**

A total of 15 patients with Federation International of Gynecology and Obsterics (FIGO) Stage IIB cervical cancer treated with cCRT were re-planned for bone marrow (BM)-sparing plans. First, we determined the optimal BM sparing strategy for intensity modulated radiotherapy (IMRT), including a BMS-IMRT plan that used total BM sparing (IMRT-BM) as the dose-volume constraint, and another plan used os coxae (OC) and lumbosacral spine (LS) sparing (IMRT-LS+OC) to compare the plan without BM-sparing (IMRT-N). Then, we determined the optimal technology for the BMS-IMRT, including fixed-field IMRT (FF-IMRT), volumetric-modulated arc therapy (VMAT), and helical tomotherapy (HT). The conformity and homogeneity of PTV, exposure volume of OARs, and efficiency of radiation delivery were analyzed.

**Results:**

Compared with the IMRT-N group, the average volume of BM that received ≥10, ≥20, ≥30, and ≥40 Gy decreased significantly in both two BM-sparing groups, especially in the IMRT-LS+OC group, meanwhile, two BMS-IMRT plans exhibited the similar effect on PTV coverage and other organs at risk (OARs) sparing. Among three common IMRT techniques in clinic, HT was significantly less effective than VMAT and FF-IMRT in the aspect of BM-Sparing. Additionally, VMAT exhibited more efficient radiation delivery.

**Conclusion:**

We recommend the use of VMAT with OC and LS as separate dose-volume constraints in cervical cancer patients aiming at reducing hematologic toxicity associated with cCRT, especially in developing countries.

## Background

Cervical cancer is the fourth most commonly diagnosed malignancy and also the fourth most frequent cause of cancer-related mortality in women worldwide (1). The cisplatin-based cCRT has been accepted as a standard treatment for most cervical cancer patients since 1999 (2). In comparison with radiation therapy (RT) alone, cCRT increases tumor control and improves patients’ prognosis (3, 4). However, the incidence of acute hematological toxicity also increases (5). Therefore, hematologic toxicity, which may result in treatment breaks and poor prognosis of patients (6–8), is a significant clinical interest during the duration of cCRT in cervical cancer patients.

Previous studies demonstrate that the occurrence of hematologic toxicity is associated with the volume of irradiated pelvic bone marrow (BM) (9–11). Therefore, BM-sparing IMRT is considered an effective strategy to reduce hematologic toxicity in pelvic IMRT (9). More recently, studies have focused on functionally active BM sparing using [^18^F] fluoro-2-deoxy-2-D-glucose (FDG) and 3’-deoxy-3’-[^18^F] fluorothymidine (FLT) positron emission tomography/CT (PET/CT) (12–15). However, functional imaging is expensive and not universally available, especially in developing countries. Therefore, we explored the optimal dose limitation strategy and radiotherapy technology in BM-sparing IMRT for cervical cancer patients based on the current RT conditions available in most developing countries.

Previous studies have shown the IMRT plans using the lumbosacral spine (LS) and os coxae (OC) as the dose-volume constraints showed the best sparing of BM (16). Currently, the radiotherapy technologies commonly used in the treatment of cervical cancer include FF-IMRT, VMAT, and HT. Therefore, the present study aims to answer 1) which BM sparing strategy (e.g., the total BM in pelvic or BM as OC and LS separately) is more beneficial BM-sparing IMRT for cervical cancer patients? and 2) which radiotherapy technology is more effective and efficient in BM-sparing IMRT for patients with cervical cancer, FF-IMRT, VMAT, or HT?

## Methods

### Patients and Imaging Data

A total of 15 patients diagnosed with FIGO Stage IIB cervical cancer and treated with cCRT in our hospital between June 1st 2019 and July 30th 2019 were selected for the present study, and patients’ information is shown in **Table 1**. All the patients undergone external beam radiotherapy (EBRT), brachytherapy, and concurrent chemotherapy. Before EBRT, all the patients were scanned using a Philips 16-slice Brilliance big bore computed tomography scanner (Philips Medical Systems, Amsterdam, Netherlands) with 5 mm slice thickness images, collected from the upper border of L2 vertebra to the region of 5 cm below the ischial tuberosities. All the patients were immobilized with thermoplastic mold in a supine position with comfortably full bladder and bowel preparation prior to simulation (after bladder emptying, patients were requested to drink 800 ml of water with 40 ml 60% Meglumine Diatrizoate 1 h before treatment and hold their urine). The CT scan images were transmitted into the Pinnacle^3^ 9.10 (Philips Medical Systems, Cleveland, USA) planning system for targets and OARs contouring. The CT scan images and RT structures were transmitted to the Monaco 5.11 (Elekta AB, Stockholm, Sweden) planning system and Tomotherapy planning system (TomoTherapy Inc., Madison, WI) for radiotherapy planning design.

**Table 1:** Clinicopathological characteristics of the patients with cervical squamous cell carcinoma.

Variables	No. Patients (*N* = 15)
Age(years)	
<45	9
≥45	6
SCC-Ag value	
1.5–10	11
>10	4
Tumor size(cm)	
≤4	10
>4	5
Deep stromal invasion	
No	4
Yes	11

### Target and Normal Tissue Delineation

Targets and OARs were delineated by the same associated chief physician. Delineation was performed according to the recommendations of the Radiation Therapy Oncology Group (RTOG) 0418 protocol and the International Commission on Radiation Units and Measurements reports (ICRU) Report 62. The clinical target volume (CTV) was defined as regions considered to embrace potential microscopic disease, including the gross tumor volume (GTV), cervix, parametria, uterus, uterosacral ligaments, sufficient vaginal margin from the gross disease (at least 3 cm), presacral region, and regional lymph nodes (e.g., common, internal, and external iliac, obturator, and presacral nodal basins). On account of the application of image guidance technique during radiotherapy, the target volume (PTV) expanded a 5 mm margin by CTV.

**Figure 1:**
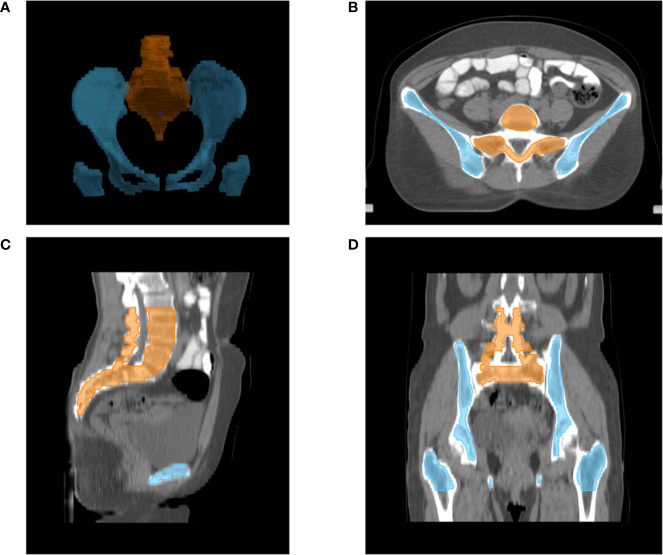
Typical figures showing contours for bone marrow cavity of pelvic: Digital reconstruction **(A)** of pelvic bone cavity of LS (orange line) and OC (blue line); An axial **(B)**, sagittal **(C)** and coronal **(D)** figure for inner cavity of LS (orange line) and OC (blue line).

The following OARs were delineated as avoidance structures: bladder, small intestine, rectum, spinal cord, femoral heads, BM, OC, LS. Total BM was contoured from the centrum 2 cm above the upper boundary of PTV to the ischial tuberosities, including the pelvis, L4-5 centrum, and sacrum. We only contoured the marrow cavity of the intramedullary low-density area, which contains most of the hematopoietically active bone marrow, based on the bone window/level of CT simulation in pinnacle 9.10 planning system. And as reported previously (16), the total BM was also divided into two parts in this study: OC—defined as the region extending from the iliac crests to the ischial tuberosities comprising the ilium, pubis, ischium, and acetabula but not containing the femoral heads; and LS—extending from centrum 2 cm above the upper boundary of PTV to the coccyx. The contour of marrow cavity was shown in **Figure 1**.

### RT Planning

All patients were treated with IMRT of 45.0 Gy in 25 daily fractions to PTV. In all plans, the PTV had the highest priority. The standard for the acceptance of the plan was that at least 95% volume of the PTV received 100% of the prescription dose, meanwhile the maximal dose of the PTV should be <110% of the prescribed dose. Dose-volume constraints for OARs were listed in **Table 2**.

**Table 2:** The dose-volume constraints of normal tissues in cervical cancer.

Structures	Dose-volume constraints
Small bowel	V40 < 30%, D_max_ < 48Gy
Rectum	V40 < 40%, D_max_ < 48Gy
Bladder	V40 < 40%
Femoral head	V40 < 5%
Spinal cord	D_max_ < 40 Gy
BM	V10 < 80%, V20 < 60%, V30 < 40%
OC	V10 < 80%, V20 < 50%, V30 < 30%
LS	V10 < 90%, V20 < 70%, V30 < 40%

### Radiotherapy Technologies

FF-IMRT plans were used to determine which between BM-sparing strategy is more beneficial in BM-sparing IMRT for patients with cervical cancer. And then we also confirmed the most effective and efficient technology among FF-IMRT, VMAT, and HT.

#### Fixed-Field IMRT Plan

The fixed-field IMRT plans were done in the Monaco 5.11 planning system and used 6 MV x-ray of a versa HD linear accelerator (Elekta Ltd., Crawley, UK). FF-IMRT plans were generated using nine evenly distributed coplanar fields with the gantry angles of 200°/240°/280°/320°/0°/40°/80°/120°/160°, and 25 control points were set in each beam. All FF-IMRT plans were calculated using Monte Carlo algorithm and the DMLC (sliding window) technique.

#### VMAT Plan

Similar to the FF-IMRT plans, the VMAT plans were done in the Monaco 5.11 planning system, used 6 MV x-ray of a versa HD linear accelerator, and computed using the Monte Carlo algorithm. The VMAT plans were designed using one beam with two full arcs, and there was 150 control points were found in each arc. The optimization objectives of the VMAT plans were the same as those of the FF-IMRT plans.

#### HT Plan

The DICOM images and RT structures were transferred to the tomotherapy planning system (Accuray Inc., Madison, WI, USA) for HT plans with 6MV photon beams. The beamlet calculation parameters included a field width of 2.5 cm, pitch value of 0.287, modulation factor of 3, and a normal dose calculation grid.

### Plan Evaluation

The dose volume histograms (DVHs) obtained from the PTV and other contoured OARs were analyzed. Dosimetric parameters, including D_98%_ (the dose received 98% volume of the PTV), D_50%_, D_2%_, the mean dose (D_mean_), conformity index (CI), and homogeneity index (HI), were quantified from the PTV. CI was defined to evaluate the conformity of prescribed dose distribution (17).

$$\text{CI}=\frac{{\text{V}}_{\text{t},\text{ref}}}{{\text{V}}_{\text{t}}}\times \frac{{\text{V}}_{\text{t},\text{ref}}}{{\text{V}}_{\text{ref}}}$$

Here V_t,ref_, V_t_, and V_ref_ denoted the target volume that receives the prescribed dose, the target volume, and the total volume covered by the prescribed dose, respectively. The CI ranges from 0 to 1, in which a higher CI represents better conformity. According to ICRU report NO.83 (18), the HI was calculated as follows:

$$\text{HI}=\frac{{\text{D}}_{2\%}-{\text{D}}_{98\%}}{{\text{D}}_{50\%}}$$

The smaller value of HI indicates better homogeneity of the target volume.

For OARs: Data analysis was carried out for the V_10_ (the OAR volume received the dose of 10 Gy), V_20_, V_30_, V_40_, D_mean_, and D_max_. Treatment time, including the time for radiation delivery and gantry rotation, was also collected and compared.

### Treatment Regimen

EBRT was delivered by FF-IMRT, VMAT, or HT, and the total dosage of EBRT was 45 Gy (applied in daily fractions of 1.8 Gy, five fractions weekly). Patients also received brachytherapy using Ir^192^ radioactivity (high-dose rate) with a total dosage of 30–36 Gy for each A point (5–6 fractions at 6 Gy per fraction) once or twice weekly. As a result of the dose in brachytherapy has a sharp decline, its dose was not included in the dosimetric analysis. Patients were also received 30–40 mg/m^2^ of cisplatin/nedaplatin once a week continuously during EBRT, beginning at the first day of radiation. The course of cCRT lasted 6–8 weeks.

### Statistical Methods

Each treatment plan was normalized to the same coverage of 95% of the PTV with the prescribed dose to maintain the comparability of the results. Then, the dose-volume histogram (DVH) parameters of PTV and OARs were analyzed using paired *t* test. Statistical analyses were performed using SPSS version 16.0 (SPSS, Chicago, IL), and *P <*0.05 was considered statistically significant.

## Results

The median age of all the included patients is 45 years (38–69 years). The mean volume values for PTV were 1,095.41 ± 135.49 cc. The mean volume values for the small intestine, rectum, and bladder were 1,471.06 ± 323.10, 64.85 ± 21.79, and 262.41 ± 148.88 cc, respectively. The mean volume value for the bone marrow was 283.34 ± 32.64 cc for LS and 436.06 ± 63.02 cc for OC, respectively.

### Comparison of IMRT-N, IMRT-BM, and IMRT-LS+OC

**Figure 2:**
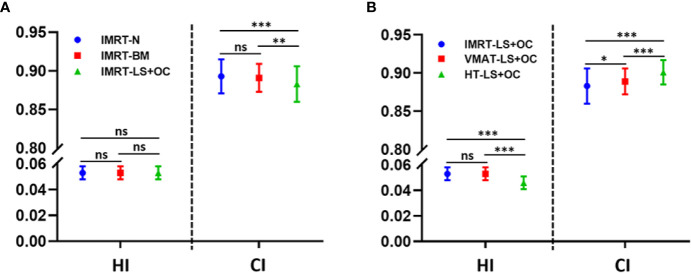
Conformity index (CI) and homogeneity index (HI) for planning target volume (PTV) with different BM-sparing strategies **(A)** and different radiotherapy technologies **(B)**. ^*^ *P* < 0.05, ^**^ *P* < 0.01, ^***^ *P* < 0.001. ns: P > 0.05, no statistical significance.

**Figure 3:**
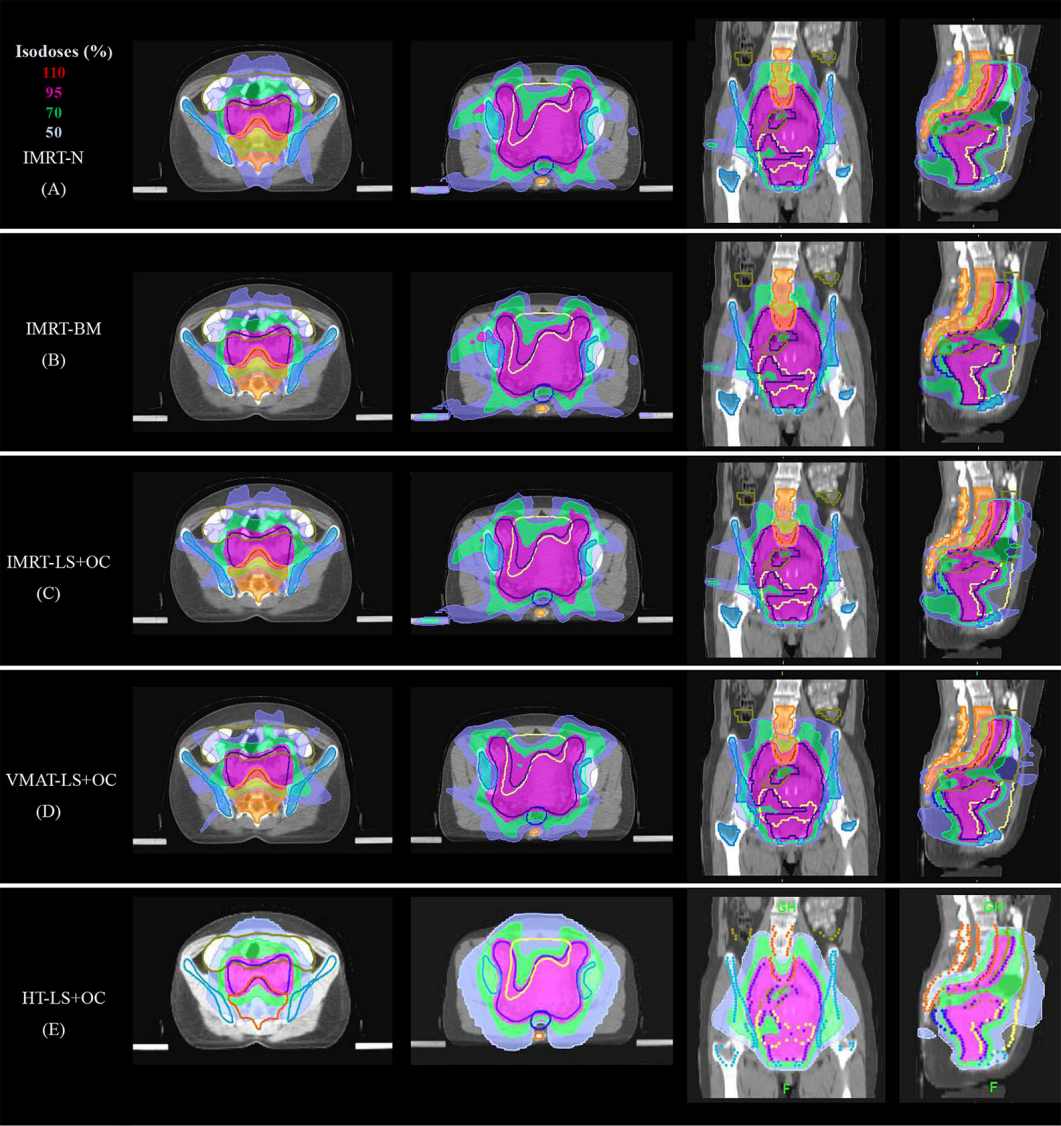
Typical dose distributions of the five plans in cervical cancer. **(A)** IMRT-N, **(B)** IMRT-BM, **(C)** IMRT-LS+OC, **(D)** VMAT-LS+OC, and **(E)** HT-LS+OC plans.

PTV dosimetric parameters and comparisons among the two BM-sparing strategies and nomal IMRT plans were summarized in **Table 3**. Compared with the IMRT-N, the IMRT-BM plans have the similar CI and HI, and the IMRT-LS+OC plans have similar HI and a slightly lower CI (**Figure 2A**), which is only approximately 1.12% (IMRT-LS+OC Vs. IMRT-N: 0.883 ± 0.023 Vs. 0.893 ± 0.022, *P* < 0.001), indicating a slightly poorer conformal dose distribution to the PTV. Also compared with IMRT-N, the mean PTV dose increased in the IMRT-LS+OC with the differences within 0.20% (IMRT-LS+OC Vs. IMRT-N: 0.883 ± 0.023 Vs. 0.893 ± 0.022, *P* < 0.001). Although a statistical difference in Dmean and CI between IMRT-N and IMRT-LS+OC was observed, the difference of the absolute value is very small and the CI in all these three types of plans is ideal. In general, all these three types of plans maintained the coverage of PTV, with 95% of the total volume receiving 100% of the prescribed dose (**Figure 3**).

**Table 3:** Dose-volume histogram comparisons for PTV and main OARs in IMRT plans.

OARs	IMRT-N	IMRT-BM	IMRT-LS+OC	P*		
				IMRT-BM VS IMRT-N	IMRT-LS+OC VS IMRT-N	IMRT-LS+OC VS IMRT-BM
PTV						
Dmean (Gy)	45.95 ± 0.11	45.96 ± 0.07	46.04 ± 0.14	0.253	0.001	0.005
HI	0.053 ± 0.005	0.053 ± 0.005	0.053 ± 0.005	0.334	1.000	0.334
CI	0.893 ± 0.022	0.891 ± 0.018	0.883 ± 0.023	0.384	<0.001	0.006
Bone marrow (BM)						
V10 (%)	86.37 ± 2.50	84.22 ± 1.48	80.99 ± 2.10	0.003	<0.001	<0.001
V20 (%)	72.30 ± 2.34	65.54 ± 2.13	58.26 ± 1.10	<0.001	<0.001	<0.001
V30 (%)	50.44 ± 3.24	43.96 ± 1.76	35.97 ± 1.32	<0.001	<0.001	<0.001
V40 (%)	21.04 ± 2.69	18.66 ± 2.04	14.71 ± 1.47	<0.001	<0.001	<0.001
Dmean (Gy)	27.92 ± 0.86	26.12 ± 0.36	24.29 ± 2.55	<0.001	<0.001	0.025
Small intestine						
V10 (%)	82.84 ± 4.60	83.04 ± 4.57	82.12 ± 4.25	0.276	0.056	0.013
V20 (%)	60.56 ± 6.29	63.43 ± 6.40	65.05 ± 5.62	<0.001	<0.001	0.027
V30 (%)	37.29 ± 7.38	37.73 ± 7.13	37.58 ± 7.37	0.164	0.528	0.745
V40 (%)	17.84 ± 5.52	18.18 ± 5.60	18.29 ± 5.44	0.007	0.026	0.513
Dmax (Gy)	47.00 ± 0.28	47.06 ± 0.19	47.16 ± 0.31	0.194	0.004	0.130
Rectum						
V10 (%)	97.35 ± 2.96	97.68 ± 2.77	97.66 ± 2.88	0.018	0.019	0.839
V20 (%)	93.73 ± 3.70	93.94 ± 3.40	93.52 ± 3.30	0.327	0.404	0.072
V30 (%)	75.97 ± 3.99	76.12 ± 3.21	74.85 ± 3.91	0.877	0.256	0.177
V40 (%)	34.14 ± 4.99	34.56 ± 5.83	35.96 ± 6.85	0.602	0.125	0.212
Dmax (Gy)	45.46 ± 0.49	45.40 ± 0.50	45.70 ± 0.52	0.481	0.013	<0.001
Bladder						
V10 (%)	98.64 ± 1.73	98.15 ± 2.08	98.19 ± 1.98	0.261	0.140	0.830
V20 (%)	82.34 ± 2.58	82.41 ± 3.36	82.41 ± 3.69	0.891	0.904	0.998
V30 (%)	63.73 ± 3.30	64.55 ± 1.93	64.57 ± 2.01	0.324	0.385	0.965
V40 (%)	43.37 ± 4.40	43.20 ± 5.71	43.51 ± 5.21	0.618	0.669	0.471
Spinal Cord						
Dmax (Gy)	34.10 ± 3.03	28.40 ± 4.88	17.70 ± 3.74	<0.001	<0.001	<0.001
Femoral head-L						
V10 (%)	76.19 ± 7.57	77.54 ± 8.25	78.96 ± 9.71	0.434	0.211	0.561
V20 (%)	38.42 ± 6.18	38.10 ± 5.55	37.61 ± 5.73	0.802	0.538	0.528
V30 (%)	14.41 ± 5.33	13.56 ± 5.23	11.74 ± 4.95	0.152	0.006	0.043
V40 (%)	0.794 ± 1.306	0.687 ± 1.290	0.687 ± 1.308	0.294	0.676	0.998
Femoral head-R						
V10 (%)	73.84 ± 8.25	75.77 ± 6.80	77.25 ± 8.59	0.356	0.034	0.351
V20 (%)	33.70 ± 5.84	33.63 ± 6.81	34.06 ± 6.52	0.945	0.790	0.615
V30 (%)	11.93 ± 5.64	11.05 ± 5.24	10.44 ± 5.59	0.130	0.019	0.236
V40 (%)	1.043 ± 1.819	0.821 ± 1.645	0.564 ± 1.416	0.401	0.199	0.097

*P value was computed by paired t test.

**Figure 4:**
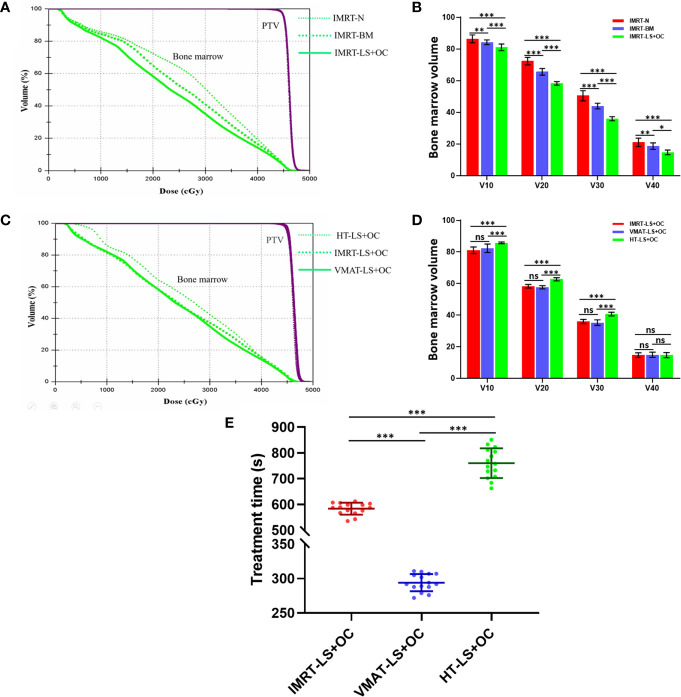
The comparison of planning target volume, bone marrow volume, and treatment time in BM-sparing IMRT. Typical dose-volume histograms for planning target volume (purple) and bone marrow (green) **(A+C)**, histogram for bone marrow volume **(B+D)** with three different dose limitation strategies for pelvic radiotherapy: IMRT-N, IMRT-BM, and IMRT-LS+OC and three different radiotherapy technologies: IMRT-LS+OC, VMAT-LS+OC, and HT-LS+OC. Typical scatter diagram for treatment time **(E)** with three different radiotherapy technologies. ^*^ *P* < 0.05, ^**^ *P* < 0.01, ^***^ *P* < 0.001. ns: P > 0.05, no statistical significance.

The dose parameters of the BM are also listed in **Table 3**, and the typical dose-volume histograms for BM are shown in **Figures 4A, B**. Compared with the IMRT-N group, the average volume of BM receiving ≥10, ≥20, ≥30, and ≥40 Gy and the mean dose decreased significantly in the two BM-sparing groups, especially in the IMRT-LS+OC group. The volume reductions are presented as follows: 2.49 and 6.23% for V10 (IMRT-BM Vs. MRT-N: 84.22 ± 1.48 Vs. 86.37 ± 2.50%, P = 0.003; IMRT-LS+OC Vs. IMRT-N: 80.99 ± 2.10 Vs.86.37 ± 2.50%, *P* < 0.001), 9.35 and 19.42% for V20 (IMRT-BM Vs. IMRT-N: 65.54 ± 2.13 Vs.72.30 ± 2.34%, *P* < 0.001; IMRT-LS+OC Vs. IMRT-N: 58.26 ± 1.10 Vs 72.30 ± 2.34%, *P* < 0.001), 12.85 and 28.69% for V30 (IMRT-BM Vs. IMRT-N: 43.96 ± 1.76 Vs. 50.44 ± 3.24%, *P* < 0.001; IMRT-LS+OC Vs. IMRT-N: 35.97 ± 1.32 Vs. 50.44 ± 3.24%, *P* < 0.001), and 11.31 and 30.09% for V40 (IMRT-BM Vs. IMRT-N: 18.66 ± 2.04 Vs. 21.04 ± 2.69%, *P* < 0.001; IMRT-LS+OC Vs. IMRT-N: 14.71 ± 1.47 Vs. 21.04 ± 2.69%, *P* < 0.001), and the mean dose reduction for bone marrow was 6.45 and 13.00% (IMRT-BM Vs. IMRT-N: 26.12 ± 0.36 Vs. 27.92 ± 0.86%, *P* < 0.001; IMRT-LS+OC Vs. IMRT-N: 24.29 ± 2.55 Vs. 27.92 ± 0.86%, *P* < 0.001).

The dose parameters of other OARs were also listed in **Table 3**. According to our results, not all the plans achieved our dose-volume constraints of OARs, the main reason is that the priority of PTV is the highest in the plan parameter setting. In the case that the OARs cannot achieve the preset conditions, the OARs will compromise to ensure the CI and HI of PTV.

The mean V20 and V40 of small intestine and V10 of rectum, were statistically and significantly increased by the IMRT-BM and IMRT-LS+OC plans compared with the IMRT-N plan. However, the difference of the absolute value is very small and still within our dose-volume constraint. On the contrary, Dmax of the spinal cord showed a significant decrease in these two BM-sparing plans, especially in the IMRT-LS+OC (IMRT-BM Vs. IMRT-N: 28.40 ± 4.88 Vs. 34.10 ± 3.03%; IMRT-LS+OC Vs. IMRT-N: 17.70 ± 3.74 Vs. 34.10 ± 3.03%, both *P* < 0.001), which may be caused by the dose limitation of the adjacent BM.

### Comparison of HT, VMAT, and FF-IMRT

Subsequently, we further investigated which radiotherapy technology is more effective in IMRT-LS+OC for patients with cervical cancer.

With regard to conformal and homogeneous dose distribution to the PTV target dose distribution, a significant benefit from the HT plans with lowest HI and highest CI were observed, and FF-IMRT and VMAT were comparable (**Table 4** and **Figure 2B**). However, HT demonstrated a more inferior sparing in the BM considering the V10, V20, and V30 of BM compared with FF-IMRT and VMAT (all *P* < 0.001) (**Figures 4C, D**). Additionally, all these three radiotherapy techniques resulted in sufficient sparing of the OARs, and we have listed all the average doses to the OARs in **Table 4**. The treatment time was also listed in **Table 4** and **Figure 4E**, the mean treatment time of FF-IMRT, VMAT, and HT were 583.53 ± 22.88, 294.13 ± 12.55, and 760.33 ± 53.48 s respectively. In comparison with conventional FF-IMRT and HT, the mean treatment time of VMAT was greatly decreased by 49.59 and 61.32% (both P < 0.001).

**Table 4:** Dosimetric parameters for PTV and main OARs in FF-IMRT, VMAT, and TOMO plans.

Parameters	FF-IMRT	VMAT	TOMO	P*		
				VMAT Vs. FF-IMRT	TOMO Vs. FF-IMRT	TOMO Vs. VMAT
PTV						
Dmean (Gy)	46.04 ± 0.14	46.05 ± 0.09	46.17 ± 0.20	0.945	0.076	0.035
HI	0.053 ± 0.005	0.053 ± 0.005	0.046 ± 0.005	1.000	<0.001	<0.001
CI	0.883 ± 0.023	0.889 ± 0.017	0.901 ± 0.016	0.014	<0.001	<0.001
Bone marrow						
V10 (%)	80.99 ± 2.10	82.30 ± 2.67	85.73 ± 0.54	0.013	<0.001	<0.001
V20 (%)	58.26 ± 1.10	57.60 ± 1.00	62.69 ± 1.00	0.061	<0.001	<0.001
V30 (%)	35.97 ± 1.32	35.27 ± 1.73	40.67 ± 1.12	0.062	<0.001	<0.001
V40 (%)	14.71 ± 1.47	14.91 ± 1.63	14.70 ± 1.63	0.450	0.956	0.334
Dmean (Gy)	24.49 ± 2.55	23.86 ± 0.27	25.56 ± 0.68	0.350	0.139	<0.001
Small intestine						
V10 (%)	82.12 ± 4.25	83.74 ± 4.77	91.45 ± 4.32	<0.001	<0.001	<0.001
V20 (%)	65.05 ± 5.62	61.82 ± 6.22	61.59 ± 6.73	<0.001	<0.001	0.758
V30 (%)	37.58 ± 7.37	37.63 ± 6.41	36.82 ± 7.74	0.894	0.137	0.118
V40 (%)	18.29 ± 5.44	18.16 ± 5.58	16.53 ± 5.39	0.460	<0.001	0.002
Dmax (Gy)	47.16 ± 0.31	47.21 ± 0.29	46.98 ± 0.32	0.541	0.06	0.005
Rectum						
V10 (%)	97.66 ± 2.88	98.28 ± 2.43	99.03 ± 1.74	0.001	0.010	0.071
V20 (%)	93.52 ± 3.30	95.30 ± 3.53	83.12 ± 7.90	<0.001	<0.001	<0.001
V30 (%)	74.85 ± 3.91	75.95 ± 3.75	54.06 ± 7.59	0.378	<0.001	<0.001
V40 (%)	35.96 ± 6.85	38.20 ± 4.57	27.26 ± 5.40	0.208	<0.001	<0.001
Dmax (Gy)	45.70 ± 0.52	43.90 ± 7.57	46.45 ± 0.53	0.360	<0.001	0.202
Bladder						
V10 (%)	98.19 ± 1.98	99.29 ± 1.25	100 ± 0.00	0.014	0.003	0.045
V20 (%)	82.41 ± 3.69	85.28 ± 5.94	92.55 ± 10.54	0.025	<0.001	0.008
V30 (%)	64.57 ± 2.01	63.39 ± 2.62	61.45 ± 5.20	0.108	0.023	0.201
V40 (%)	43.51 ± 5.21	42.38 ± 5.45	35.89 ± 4.50	0.039	<0.001	<0.001
Spinal Cord						
Dmax (Gy)	17.70 ± 3.74	13.98 ± 2.32	22.42 ± 3.75	<0.001	0.006	<0.001
Femoral head-L						
V10 (%)	78.96 ± 9.71	86.80 ± 10.09	98.67 ± 2.00	0.005	<0.001	0.001
V20 (%)	37.61 ± 5.73	29.48 ± 8.31	66.52 ± 9.96	0.002	<0.001	<0.001
V30 (%)	11.74 ± 4.95	4.98 ± 3.30	29.41 ± 11.35	<0.001	<0.001	<0.001
V40 (%)	0.687 ± 1.308	0.089 ± 0.204	0.235 ± 0.593	0.078	0.221	0.349
Femoral head-R						
V10 (%)	77.25 ± 8.59	82.96 ± 7.79	98.92 ± 1.99	0.043	<0.001	<0.001
V20 (%)	34.06 ± 6.52	26.89 ± 12.49	66.22 ± 9.23	0.064	<0.001	<0.001
V30 (%)	10.44 ± 5.59	4.29 ± 5.32	25.72 ± 10.61	<0.001	<0.001	<0.001
V40 (%)	0.564 ± 1.416	0.066 ± 0.144	0.058 ± 0.201	0.161	0.190	0.888
Treatment time (s)	583.53 ± 22.88	294.13 ± 12.55	760.33 ± 53.48	<0.001	<0.001	<0.001

*P value was computed by paired t test.

## Discussion

Our study explored the optimal bone marrow sparing strategy and radiotherapy technology in BM-sparing IMRT for cervical cancer patients who receive the cCRT. Our results demonstrated that BM-sparing IMRT plan with OC and LS as separate dose-volume constraints achieved the best BM-sparing without compromising target volume dose coverage and increasing the radiation dose to other OARs. We also demonstrated that all techniques achieved adequate coverage of PTV, however, VMAT planning yielded better BM-Sparing and shorter estimated treatment times when compared to HT planning.

It is known that BM is composed of both hematopoietically active “red” marrow and inactive “yellow” marrow, and approximately 50% of hematopoietically active BM lies within the lumbar sacrum, ilium, ischium, pubis, and proximal femur (19), where are just adjacent to the pelvic EBRT field of cervical cancer. Meantime, BM is extremely sensitive to irradiation, haematopoietic stem cells is damaged and BM microenvironment is modified adversely even at low doses (20–23). Previous studies indicated that the mean radiation dose and the volume that receives lower dose irradiation of the pelvic bone marrow were relative to hematologic toxicity (24–27). With the development of radiotherapy technology, IMRT that uses multiple beam angles or arcs, can reduce irradiation dose to OARs, which is correlated with reduced acute and late adverse events (AEs) (28–30). However, the irradiation of a large volume of BM is unavoidable during pelvic IMRT.

As most of previous studies focusing on PET-guided functional BM demonstrated that most of the functional BMs were located in the marrow cavity (12–15), OC, LS, and total BM were delineated on the basis of the marrow cavity of the intramedullary low-density area in our current study considering limited medical resources and treatment cost in our developing countries. Our results demonstrated that BM-sparing IMRT plan with OC and LS as separate dose-volume constraints achieved the best BM-sparing, with the lowest volume of BM receiving ≥10, ≥20, ≥30, and ≥40 Gy. With the rapid development of the field of artificial intelligence, the program of automatic delineation of OARs is becoming mature. We applied the program of automatic delineation of OARs in the MIM version 6.7 system (MIM Software, Cleveland, USA) through a database of 50 patients and delineated the OC and LS relatively simple. Therefore, we recommended to introduce OC and LS as the independent OARs and use dose-volume constraints in BM-sparing IMRT. These results were similar to previous study on BM-IMRT in cervical cancer of Prof. Bao (16), and the main innovations of our study are the delineation method of bone marrow and the dose constraint method of LS and OC.

Highly conformal radiation techniques have dramatically improved, in which the most representative ones are HT and VMAT. Compared with FF-IMRT plan, HT plan has a highly conformal dose distribution and reduces the high-dose volume of OARs close to the target (31, 32) while VMAT plans have less monitor units (MUs) and treatment time. Our results from the dosimetric comparison of these technologies in BM-sparing IMRT demonstrated that all plans met our clinical demand, and HT demonstrated the highest CI and lowest HI but the inferior sparing of low-dose radiation in BM (e.g., V10, V20, and V30). Meanwhile, compared with FF-IMRT and HT, the main advantage of VMAT was that it spent relatively less time on radiation delivery, thereby reducing patients’ discomfort and the probability of patents’ moving during treatment. Collectively, these results indicate that VMAT can be considered the most optimized and effective technology in BM-sparing IMRT of cervical cancer. To the best of our knowledge, no published data compare the planning parameters of HT and VAMT vs. FF-IMRT in terms of the effectiveness and efficiency of BM-sparing IMRT in cervical cancer patients.

As we know, the medical resources are insufficient in most of the developing countries. Barriers to availability of radiotherapy in these countries are attributed primarily to costs, including equipment cost and the cost of staff education. There are about only two medical linear accelerators per million people in China. Our results demonstrated that in the BM-sparing IMRT, the mean treatment time of VMAT was greatly decreased by 49.59 and 61.32%, compared with the conventional FF-IMRT and HT. The results are consistent with previous studies in other solid cancers (33–35). Therefore, VMAT, with a better dose conformity or sparing of OARs and a shorter treatment delivery time, is more recommended in the BM-sparing IMRT for cervical cancer patients, especially in the developing countries.

Our study has some limitations. First, we generally contoured the low-density regions of the bone (marrow-cavity) as the BM based on the bone window/level of CT simulation in pinnacle 9.10 planning system, this strategy may overestimate the volume of active BM or define the active BM inadequately. Second, we used the choice of uniform outward expansion of 5mm from CTV to generate a PTV in all the cases because we used image-guided technique to reduce the positioning error. However, due to the influence of organ motion, positioning error and other factors, the generation rules of PTV of radiotherapy for cervical cancer have not reached a consensus. Lastly, this study only focused on the dosimetric advantages of BM-sparing IMRT technology at the planning and design level. Future work should focus on its clinical significance correlated with the hematologic toxicity of cervical cancer patients treated with cCRT.

## Conclusion

Our study confirmed that BM-sparing IMRT plan with OC and LS as separate dose-volume constraints achieved the best BM-sparing without compromising target volume dose coverage. Moreover, VMAT exhibited the optimal BM-sparing and efficiency in all the advanced radiotherapy technologies. Therefore, VMAT with OC and LS as separate dose-volume constraints, as an effective BM-sparing IMRT, is a promising treatment option to reduce hematologic toxicity for cervical cancer patients in developing countries with limited medical resources and expenditures, not only avoids expensive functional imaging of active bone marrow but also improves the efficiency of radiotherapy.

## Data Availability Statement

The original contributions presented in the study are included in the article/supplementary material. Further inquiries can be directed to the corresponding authors.

## Ethics Statement

This study complied with the Helsinki Declaration and approval from the Ethics Committee of Harbin Medical University Cancer Hospital (Harbin, China) was obtained. All patients provided their written informed consent for the publication of their images/data.

## Author Contributions

S-SY, D-YY, and Y-YZ designed the study. S-SY contoured the target and OARs. D-YY performed the design of the treatment planning. W-KY, YF, LW, J-YS, and XL collected the data. D-YY, YF, LW, Y-YZ, and S-SY wrote and revised the manuscript. Y-LB and Y-YZ polished the language. All authors contributed to the article and approved the submitted version.

## Funding

This study was supported by grants from the National Natural Science Foundation of China (No. 81872460 and 81602664), Project of precise radiotherapy spark program (2019-N-11-11), and Pandeng Project of National Cancer Center of China (NCC201808B016).

## Conflict of Interest

The authors declare that the research was conducted in the absence of any commercial or financial relationships that could be construed as a potential conflict of interest.
